# Editorial Notes

**Published:** 1924-04

**Authors:** 


### Editorial Notes.

**Deaths of Dr. A. J. Harrison and Mr. R. G. Poole Lansdown.**

In this issue we have to record simultaneously the death of two distinguished past Presidents of the Society, Dr. A. J. Harrison and Mr. R. G. Poole Lansdown, of whom portraits and appreciation of their work are included.

Dr. Harrison masked his well advanced years by his remarkable mental and physical activity up to the day of his accident while running to catch a bus. There was much to record, but we observe no mention made of the very active part he took in the foundation of the Gloucester, Somerset and Wilts branch of the National Society for Prevention of Tuberculosis, which was initiated in 1899, and in the building of the Winsley Sanatorium which was the outcome of this three counties branch.

Mr. R. G. Poole Lansdown was a very successful consulting surgeon, but perhaps the most permanent record of his manifold professional and other activities lies in the reconstruction and development of the Chesterfield Nursing Home, a scheme which is the work of many but mainly due to Mr. Lansdown and the Hon. Treasurer, Mr. Hedley Hill. We are glad that Mr. Lansdown lived to see this complete and handsome block of buildings opened last summer, and to continue planning for its further development up to the day of his sudden death—at a time when he was in full activity and his surgical skill unimpaired. When the plans are fully developed and complete the Medical Profession in Bristol should find in it all the resources of a thoroughly well-equipped paying hospital, and Lansdown's share in bringing about this happy consummation should ever be remembered.

### MEETINGS OF SOCIETIES.

